# Venoarterial Extracorporeal Membrane Oxygenation for Severe Neonatal Acute Respiratory Distress Syndrome in a Developing Country

**DOI:** 10.3389/fped.2020.00227

**Published:** 2020-05-28

**Authors:** Xiaoyang Hong, Zhe Zhao, Zhenqiu Liu, Change Liu, Jie Wang, Xueli Quan, Hui Wu, Qiong Ji, Jianwei Sun, Donglinag Cheng, Zhichun Feng, Yuan Shi

**Affiliations:** ^1^Pediatric Intensive Care Unit, Affiliated Bayi Children's Hospital, The Seventh Medical Center, PLA General Hospital, Beijing, China; ^2^The Second School of Clinical Medicine, Southern Medical University, Guangzhou, China; ^3^Department of Neonatology, Children's Hospital, Chongqing Medical University, Chongqing, China; ^4^Surgical Pediatric Intensive Care Unit, Children's Hospital Affiliated of Zhengzhou University, Zhengzhou, China; ^5^Department of Neonatology, The First Hospital of Jilin University, Changchun, China; ^6^Department of Neonatology, Henang Provincial People's Hospital, Zhengzhou, China

**Keywords:** extracorporeal membrane oxygenation, neonatal acute respiratory distress syndrome, propensity score analysis, venoarterial mode, developing countries

## Abstract

**Objective:** Extracorporeal membrane oxygenation (ECMO) has supported oxygen delivery and carbon dioxide removal in neonatal severe respiratory failure for more than 4 decades. The definition and diagnosis of neonatal acute respiratory distress syndrome (ARDS) was made according to the criteria first established by a Montreux Conference in 2017. By far, there has been no ECMO efficiency studies in neonatal ARDS. We aimed to compare the outcomes of neonates with severe ARDS supported with and without ECMO.

**Design:** Retrospective pair-matched study.

**Setting:** In the present retrospective pair-matched study, the outcomes of severe ARDS with ECMO support and without ECMO support were analyzed and compared. Propensity score matching was conducted. The study subjects were selected from a China Neonatal ECMO (CNECMO) study. In total, five hospitals were included in the CNECMO study. The patients were matched with demographic and clinical data. The primary endpoint was in-hospital mortality. Secondary outcomes included ventilator-time, ICU stay, hospitalization costs and cranial MRI results.

**Patients:** 145 neonates with severe ARDS (Oxygenation Index, OI ≥16) from 5 hospitals.

**Interventions:** No interventions.

**Measurements and Main Results:** We collected the data of 145 neonates with severe ARDS (Oxygenation Index, OI≥16) from 5 hospitals. Among them, 42 neonates received venoarterial (VA) ECMO support, and the remaining 103 neonates were treated with conventional mechanical ventilation. The mortality of ECMO-supported neonates was not significantly different compared with the ESLO neonatal respiratory-supported from 2012 to 2018 (23.8 vs. 32.5%, *p* = 0.230). After matching with the propensity score we got 31 pairs. The ECMO-supported neonates had a lower in-hospital mortality (6 of 31, 19.4%) vs. non ECMO-supported patients (18 of 31, 58.1%) (*p* = 0.002). Hospitalization costs of survivors in ECMO-supported neonates were significantly higher than that of non-ECMO-supported neonates (*p* < 0.001). There was no difference of ventilator-times (*p* = 0.206), ICU stay (*p* = 0.879) and cranial MRI (*p* = 0.899) between the survivors of ECMO-supported and non–ECMO-supported neonates with ARDS.

**Conclusions:** By far, there has been no ECMO efficiency studies in neonatal ARDS. This study found that ECMO-support have superior outcomes compared with non–ECMO-support in neonates with severe ARDS.

## Key Points

In neonates with severe ARDS (OI≥40), our results demonstrate that ECMO-supported neonates have superior outcomes compared with non-ECMO-supported neonates, and didn't prolong ventilator-time or ICU stay.

## Introduction

Over 40 years ago, Dr. Robert Bartlett saved a neonate, Esperanza, who had severe meconium aspiration syndrome (MAS) and was failed by conventional medical therapy, with extracorporeal membrane oxygen (ECMO) (1). Evidence of the outcome benefit of ECMO for neonatal hypoxic respiratory failure (HRF) was provided by clinical trials performed in the United Kingdom (2–4). Cochrane systematic reviews of this evidence demonstrated that ECMO has been used for rescuing HRF with a survival advantage (5–7). According to the Extracorporeal Life Support Organization (ELSO), ~800 neonates worldwide receive ECMO support for respiratory failure each year (8). Although the neonatal acute respiratory distress syndrome (ARDS) has been present in clinical practice for a long time, no expert consensus has been reached regarding the definition of it. The Pediatric Acute Lung Injury Consensus Conference (PALICC) has described the definition, diagnosis and treatment of pediatric ARDS, which cannot be applied in neonates (9–11). In 2017, the Montreux definition of neonatal ARDS was introduced; neonatal respiratory failure included ARDS, and other primary diseases such as congenital diaphragmatic hernia (CDH) and idiopathic persistent pulmonary hypertension of the newborn (PPHN) with “black lung” were isolated (12). In 2018, the ECMO to Rescue Lung Injury in Severe ARDS (EOLIA) trial found no evidence of ECMO-support reducing the mortality of adult ARDS as compared with conventional ventilation (13). Moreover, another study revealed that no superior outcome in the ECMO group of pediatric ARDS was found compared to the non-ECMO group (14). How about the outcome of ECMO support in neonatal ARDS? The aim of our study is to determine whether ECMO-support has a superior outcome compared with non–ECMO-support in neonates with ARDS.

## Methods

### Study Design and Participants

The retrospective pair-matched study collected data from the China Neonatal ECMO (CNECMO) study. From 2013 to 2018, 5 hospitals in China were included. Institutional review board approval was obtained in each center. This study was registered at https://www.clinicaltrials.gov; Identifier: NCT03607760 (clinicaltrials.gov; Identifier: NCT03607760).

According to the Montreux definition of ARDS, one hundred and forty-five neonatal patients who met the criteria of severe ARDS (Oxygen Index, OI≥16) had been enrolled (12). All the neonates needed intubation for invasive mechanical ventilation support.

In the present study, we included retrospective data from neonates with severe ARDS (OI≥16), and the remaining neonates with non-ARDS respiratory failure were excluded (12). ECMO was initiated in neonates with OI≥40 for 4 h or in neonates with right ventricular dysfunction that failed to respond to maximal care after informed consent of the parents was obtained (15, 16). And patients without ECMO indications were excluded. This included neonates with lethal chromosomal disorder (trisomy13 and 18), irreversible brain damage, Grade III or greater intraventricular hemorrhage, <2 kg and <34 weeks (17).

The neonates with ECMO indications and with non-ECMO support due to failure of parent consent were treated with maximal intensive care, including the lung protective ventilator settings, HFOV, prone position ventilation, PS, NO, restricted fluid intake, sedation and analgesia. Patients without ECMO were treated with conventional mechanical ventilation (inspiratory pressure above PEEP of 16–20 cmH_2_O, PEEP of 4–10 cmH_2_O, FiO_2_ of 60–98% and RR of 35–50/min) or with HFOV if the mean airway pressure was over 12 cmH_2_O. Once the neonate was supported with HFOV, the ventilation of HFOV would be set as follows: mean airway pressure of 13–18 cmH_2_O, Amplitude of 4–6, Rate of 8–10 Hz and FiO_2_ of 60–98%.

### Neonatal ECMO Management

ECMO support was provided in 2 centers. Both of the centers ran ECMO cases over 20 cases per year. All neonatal ECMO teams including nurses, perfusionists, cardiac surgeons, neonatologists, and intensive care pediatricians were trained according to the ELSO guidelines. The two ECMO centers followed the same ECMO inclusion criteria and instruction for running as discussed, and got an agreement before the study. VA-ECMO and a centrifuge pump was put on all of the ECMO-supported neonates. Once the patient was stabilized on ECMO support, the ventilator was set on the “rest mode.” Peak inspiratory pressure (PIP) of 15–22 cmH_2_O, a positive end-expiratory pressure (PEEP) of 5–8 cmH_2_O, a rate of 10–20/min, an inspiratory time of 0.5 s, and a FiO_2_ of 0.21–0.3 were adopted (15).

### Data Collection

All data including demographic data, diagnosis, ventilator settings, blood-gas analysis, oxygenation index (OI) and arterial lactate values were recorded after hospital admission. In ECMO-supported neonates, extra data were also recorded after the initiation of ECMO.

### Outcomes

The primary outcome was in-hospital mortality. Secondary outcomes included ICU stay, ventilator-time, hospitalization costs and cranial MRI results.

### Statistical Analysis

Clinical characteristics of all neonates were analyzed and compared with generalized estimating equations as a cluster variable for matching. Then, propensity score matching was performed for the pair-matched cohort analysis on the basis of variables expected to be associated with the use of ECMO. As a result, a 1:1 matching pair was made without replacement on a basis of daily observation. The first ECMO day was the exact observation day for the ECMO-support neonates. However, for those non ECMO-support neonates, several days during their courses of disease could be eligible for matching. So, each combination of patient-day was compared separately. The median of continuous and ordinal variables and exact conditional maximum likelihood estimation of binary variables were calculated. The characteristics and outcomes of each pair were compared based on the exact sign tests and 95% confidence intervals for the median and estimation.

Continuous OI and primary diagnosis were calculated as the covariates for the multivariable logistic regression. The regression was used for estimation of the probability of ECMO treatment to get a propensity score matching. After that, the smallest difference in the propensity scores was the standard for each ECMO and non-ECMO supported paired observation.

## Results

**Figure 1:**
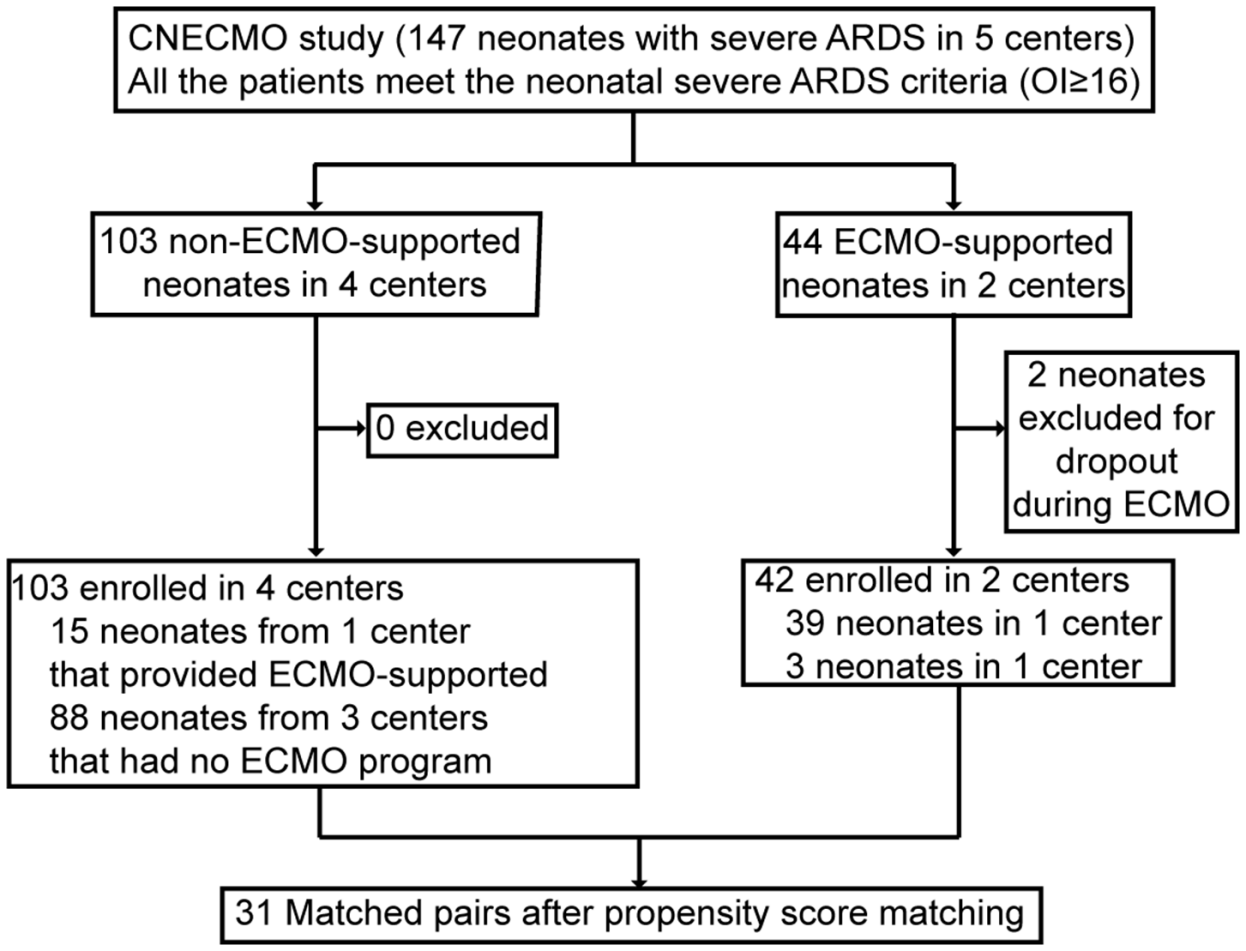
Flow chart for the exclusion and matching of subjects in China neonatal ECMO study.

Forty-four neonates received VA-ECMO for severe ARDS at 2 of 5 participating centers (40%). Two cases were excluded from the analysis, because they left NICU during ECMO support. Because double-lumen cannula has not been registered in the People's Republic of China, the venoarterial ECMO cannulation strategy was used in all neonatal ECMO cases. A hundred and three non-ECMO-supported neonates with severe ARDS criteria met inclusion criteria from 4 participating centers (OI≥16). Of whom, 88 were cared for at 3 centers that did not have a neonatal ECMO program, and 15 of them were from the center that provided ECMO support. Among these 15 neonates, 13 neonates with OI≥40 did not receive ECMO support, because their parents refused ECMO, mainly concerned of the cost (Figure 1). Among the 13 neonates, 7 died of hypoxemia and the other 6 survived. One of the survivors got intracranial hemorrhage and another one's cranial MRI showed hypoxic ischemic encephalopathy. In both groups, the primary cause of death included poor recovery of the lung and died of hypoxemia directly. The other causes of death in the ECMO supported neonates were intracranial hemorrhage, irreversible bloodstream infection and associated sepsis. Among neonates without ECMO support, besides hypoxemia, ventilator associated pneumonia was the second cause of death and followed by DIC.

Before matching, ECMO-supported neonates had higher OI, PaCO_2_ and lactate levels, lower PH and BE compared with non-ECMO-supported neonates pre-ECMO support, suggesting a higher risk of mortality of ECMO-supported neonates at admission. The primary diagnosis was totally different between ECMO and non-ECMO-supported neonates, MAS was the major diagnosis in ECMO-supported neonates (40.5%), and pneumonia was the major diagnosis in non-ECMO-supported neonates. Higher use of NO inhalation and lower use of HFOV were found in ECMO-supported neonates. No differences were shown between two groups of neonates in age at admission, gestational age (GA), body weight, arterial PaO_2_ pre-ECMO-support, sex, delivery and PS (Table 1).

**Table 1:** Characteristics of ECMO-supported and non-ECMO-supported neonates with severe ARDS.

**Characteristics**	**ECMO (*n* = 42)**	**Non-ECMO (*n* = 103)**	***P*-value**
**BASIC INFORMATION**			
Age at admission (h), median (IQR)	6.75 (5~13.25)	8 (4~15)	0.99
GA (Weeks), median (IQR)	39 (37^+5^~40)	39^+4^ (38^+1^~40^+3^)	0.32
Body weight (g)	3,362 ± 487.73	3243.4 ± 542.61	0.221
Sex *n* (%)			0.216
Male	31 (73.8%)	65 (63.1%)	
Female	11 (26.2%)	38 (36.9%)	
Delivery, *n* (%)			0.145
Spontaneous labor	15 (35.7%)	23 (23.7%)	
Cesarean delivery	27 (64.3%)	74 (76.3%)	
Diagnosis, *n* (%)			0.002^**^
MAS	17 (40.5%)	19 (18.4%)	
Sepsis	8 (19.0%)	10 (9.7%)	
Pneumonia	11 (26.2%)	61 (59.2%)	
Asphyxia	6 (14.3%)	13 (12.6%)	
**ILLNESS SEVERITY METRICS PRE-ECMO**			
OI, median (IQR)	51.2 (42.2~63.9)	25 (18.8~35.958)	<0.001^**^
PH, median (IQR)	7.2 (7.1~7.3)	7.3 (7.2~7.4)	0.024^*^
PaO_2_, median (IQR)	34.9 (30~41.2)	34 (28.9~46)	0.794
PaCO_2_, median (IQR)	53.0 (39.5~61.5)	42.8 (37~52)	0.012^*^
BE, median (IQR)	−9.9 (−15.5~-2.5)	−5.1 (−7.7~-1.9)	0.009^**^
Lactate, median (IQR)	8.8 (4.8~14.9)	4.2 (2.8~5.5)	<0.001^**^
Treatment pre-ECMO			
NO, *n* (%)	29 (69.0%)	41 (39.8%)	0.001^**^
PS, *n* (%)	36 (85.7%)	88 (85.4%)	0.966
Ventilator mode, *n* (%)			<0.001
HFOV	24 (57.1%)	86 (84.3%)	
CMV	18 (42.9%)	16 (15.7%)	

ECMO, extracorporeal membrane oxygenation; IQR, interquartile range; GA, gestational age; MAS, meconium aspiration syndrome; OI: oxygenation index; BE, base excess; NO, nitrix oxide; PS, pulmonary surfactant; HFOV, high frequency oscillatory ventilation; CMV, conventional mechanical ventilation;

* P < 0.05;

** *P < 0.01*.

According to the propensity score matching approach and excluding thirteen cases with imputed data, 31 matched pairs were got. After matching, covariates reached a good balance for all matched variables and the results were shown in Table 2. ECMO-supported neonates had slightly higher PaO_2_ and lactate levels pre-ECMO support.

**Table 2:** Characteristics of ECMO-supported and non-ECMO-supported neonates with severe ARDS after propensity score matching.

**Characteristics**	**ECMO (*n* = 31)**	**Non-ECMO (*n* = 31)**	***P*-value**
**BASIC INFORMATION**			
Age at admission (h), median (IQR)	7 (5~14)	5 (4~10)	0.408
GA (Weeks), median (IQR)	39^+3^ (38^+1^~40^+5^)	39^+1^ (38~40)	0.607
Body weight (g)	3364.84 ± 472.05	3285.81 ± 560.69	0.551
Sex, *n* (%)			0.118
Male	22 (71.0%)	16 (51.6%)	
Female	9 (29.0%)	15 (48.4%)	
Delivery, *n* (%)			0.788
Spontaneous labor	11 (35.5%)	10 (32.3%)	
Cesarean delivery	20 (64.5%)	21 (67.7%)	
Diagnoses, *n* (%)			0.149
MAS	12 (38.7%)	8 (25.8%)	
Sepsis	6 (12.9%)	2 (6.5%)	
Pneumonia	8 (25.8%)	16 (51.6%)	
Asphyxia	5 (16.1%)	5 (16.1%)	
**ILLNESS SEVERITY METRICS PRE-ECMO**			
OI, median (IQR)	46.3 (40.6~62.5)	41 (35.2~62.5)	0.147
PH, median (IQR)	7.24 (7.08~7.33)	7.20 (7.12~7.33)	0.959
PaO_2_, median (IQR)	36 (30~47)	29 (22~32)	0.019^*^
PaCO_2_, median (IQR)	53.0 (39.7~59)	45 (36~59)	0.408
BE, median (IQR)	−7.4 (−15.7~-2.5)	−6.6 (−9.5~-3.4)	0.253
Lactate, median (IQR)	7.7 (4.4~13.2)	2.6 (4.67~6.8)	0.04^*^
**Treatment PRE-ECMO**			
NO, *n* (%)	20 (64.5%)	13 (41.9%)	0.075
PS, *n* (%)	26 (83.9%)	27 (87.1%)	1
Ventilation, *n* (%)			0.168
HFOV	19 (61.3%)	24 (77.4%)	
CMV	12 (38.7%)	7 (22.6%)	

ECMO, extracorporeal membrane oxygenation; IQR, interquartile range; GA, gestational age; MAS, meconium aspiration syndrome; OI: oxygenation index; BE, base excess; NO, nitric oxide; PS, pulmonary surfactant; HFOV, high frequency oscillatory ventilation; CMV, conventional mechanical ventilation;

* defined as P < 0.05;

** *defined as P < 0.01*.

In propensity score-matched neonates, the hospital mortality rate was 19.4% for ECMO supported vs. 58.1% for non–ECMO supported patients (*P* = 0.002). The ventilation times were 264 h (interquartile range [IQR], 192–352 h) for the ECMO-supported ones vs. 112 h (48–192 h) for the non–ECMO-supported neonates (*P* < 0.001). The ICU stay was 21 days (IQR, 15-29 days) for the ECMO-supported vs. 6 days (3–24 days) for the non–ECMO-supported neonates (*P* < 0.022). The hospitalization costs were 24,705.9USD (IQR, 18,235.3~28,529.4USD) for the ECMO-supported vs. 4,705.9USD (3,235.3~7,500.0USD) for the non–ECMO-supported neonates (*P* < 0.001) (Table 3). In the ECMO group, the severity of OI between the survivors and dead neonates showed no significant difference (*P* = 0.455).

**Table 3:** The primary and secondary outcomes analyzed by propensity score matching.

**Outcomes at discharge**	**ECMO**	**Non-ECMO**	**OR or Median of differences**	**95%CI**	***P*-value**
	**(*n* = 31)**	**(*n* = 31)**			
Survival, *n* (%)	25 (80.6%)	13 (41.9%)	5.77	(1.84~18.06)	0.002^**^
Mortality, n (%)	6 (19.4%)	18 (58.1%)			
Ventilator-time (h), median (IQR)	264 (192~352)	112 (48~192)	158	(48~207)	<0.001^**^
ICU stay (d), median (IQR)	21 (15~29)	6 (3~24)	12	(2~16)	0.022^*^
Hospital costs (Dollar), median (IQR)	24705.9 (18235.3~28529.4)	4705.9 (3235.3~7500)	12.34	(15882.4~21029.4)	<0.001^**^

ECMO, extracorporeal membrane oxygenation; OR, odds ratio; IQR, interquartile range; CI, confidence interval;

* P < 0.05;

** *P < 0.01*.

Among the survivors of 31 propensity-matched, before the patients left the ICU, the evaluation of both carotid artery and jugular vein was done by the ultrasound. Each survivor would receive a cranial MRI before they discharged, for the parents and their families were all concerned about the state of the central nervous system. No difference was observed between the survivors of the two groups with ARDS in ventilator-time (*p* = 0.206), ICU stay (*p* = 0.879) and cranial MRI (*p* = 0.899) (Table 4).

**Table 4:** The Secondary outcomes analyzed in survivors by propensity score matching.

**Secondary outcomes**	**ECMO**	**Non-ECMO**	**OR or Median of differences**	**95%CI**	***P*-value**
	**(*n* = 25)**	**(*n* = 13)**			
Ventilator-time (h) of survivors, median (IQR)	288 (214~320)	271 (168~297.5)	17	(−61~120)	0.206
ICU stay of survivors(d), median (IQR)	21 (18~28.5)	22 (17~28)	−1	(−7~9)	0.879
Hospital costs of survivors (Dollar), median (IQR)	24482.4 (20720.6~27161.8)	8294.1 (4720.6~11691.2)	11.2	(11441.2~20911.8)	<0.001^**^
Normal cranial MRI of survivors, *n* (%)	14 (56%)	7 (53.8%)	1.09	(0.28~4.19)	0.899

ECMO, extracorporeal membrane oxygenation; OR, odds ratio; IQR, interquartile range; CI, confidence interval;

* P < 0.05;

** *P < 0.01*.

In total, 42 neonates with ARDS received ECMO support, and the mortality was 23.8%. The mortality of neonates with ECMO support for respiratory failure in ELSO from 2013 to 2018 was 32.5%, and there were no significant differences of mortality (*p* = 0.230) between the CNECMO and ELSO (Table 5).

**Table 5:** The outcomes of CNECMO compared with ELSO (2012~2018).

	**Survival,** ***n* (%)**	**Non-survival,** ***n* (%)**	***P*-value**
CNECMO (*n* = 42)	32 (76.2)	10 (23.8)	0.23
ELSO (*n* = 4112)	2775 (67.5)	1337 (32.5)	

CNECMO, China neonatal extracorporeal membrane oxygenation; ELSO, extracorporeal life support organization;

* P < 0.05;

** *P < 0.01*.

## Discussion

In this study of 31 propensity-matched neonates with severe ARDS supported with and without ECMO, we found significantly lower in-hospital mortality in the ECMO-supported group. Neonatal ARDS is a new conception, according to the Montreux definition in 2017 (12). Not all neonatal respiratory failure can be diagnosed as neonatal ARDS. To our knowledge, this is the first study in neonatal ARDS with or without ECMO support. For neonates with ARDS, few clinical trials have published about mortality comparing between ECMO-support and non ECMO-support patients. Our study attempts to determine ECMO efficacy by analyzing the rigorously collected data of 5 centers in China. The 103 non–ECMO-supported neonates with severe ARDS offered enough cases for balance matches.

In our study, the neonates with severe ARDS were treated with ECMO support according to the ELSO guideline (2). Among all the factors associated with the decision to be on ECMO, OI was the most important indication. OI, use of NO, PS and HFOV were similar, suggesting that the threshold is the same for initiating ECMO support in neonates with ARDS. It is practicable for comparison between the ECMO-support and non ECMO-support neonates with the propensity matched cohort study. Before matching, the base-line of clinical characteristics from both groups were quite different. OI is an important index, evaluating the severity of respiratory failure in clinical practice. It is closely associated with prognosis: the higher the OI, the higher the risk of mortality in ECMO-supported neonates before propensity matched (14, 18, 19). A higher use of HFOV in non-ECMO-supported than ECMO-supported neonates was observed, probably due to HFOV being a remedy choice for neonates with severe ARDS in hospitals without ECMO equipment. After propensity score matching, we found that the basic clinical characteristics between the two groups were without significant differences. Although PaO_2_ in ECMO-supported neonates were higher than that of the non-ECMO-supported neonates, PH and OI, indexes indicating disease severity and prognosis were without significant differences. The lactate levels in ECMO-supported remained higher than that of the non-ECMO-supported neonates pre-ECMO support, suggesting that neonates in the ECMO-supported group were probably more critically ill than neonates in the non-ECMO supported group. Lactate levels and lactate clearance had been proven to be predictors of mortality in adult patients with ECMO support (20, 21). Nonetheless, after propensity matching that OI value and the primary disease causing neonatal ARDS matched, hospital mortality of ECMO-supported neonates (19.4%) was markedly lower than that of the non-ECMO supported neonates (58.1%). For neonatal ARDS with OI≥40, ECMO support profoundly reduced their mortality, indicating that for neonatal ARDS, the indication of ECMO support, OI≥40, might be too strict in that some critically ill neonates with ARDS could be denied the chance of ECMO support, and thus increasing the incidence of mortality.

The 42 neonates receiving ECMO in this study had a similar mortality. Compared to neonates reported in the ELSO Registry between 2013 and 2018 (23.8 vs. 32.5%, *p* = 0.23) (22), mortality was lower in the present study, due to the fact that ECMO-supported neonates in the present study didn't include CDH neonates. The neonates who need ECMO support for CDH have the worst outcomes among all the neonatal respiratory failure diseases (16, 23, 24). There was no difference in ventilator-time or ICU stay among the survivors between the two groups. For neonates with severe ARDS (OI≥40), ECMO support decreased mortality, without prolonged ventilator-time or ICU stay. On the contrary, the famous clinical trial, CESAR (Conventional Ventilation or ECMO for Severe Adult Respiratory Failure), revealed a longer ICU and hospital length of stay for adult patients randomized to receive ECMO treatment in an ECMO center (25). Hospital costs were significantly higher in the ECMO-supported compared with non-ECMO-supported of 31 propensity-matched neonates, and the same if restricted to survivors. ECMO costs were not covered by the insurance system of China. It is challenging for parents to pay the cost of ECMO. Some neonates with ARDS who met neonatal respiratory ECMO indications gave up ECMO support because of financial difficulty.

Considering the possible central nervous damage caused by the ligation of carotid artery, cranial MRI were carried out before the newborn babies' discharge. And no difference was found between the two groups. Some studies have proved that the risk of neurologic injury is higher in VA-ECMO and the mortality is lower and the length of bypass shorter in VV-ECMO (17, 26–28). In our study, when VA-ECMO was removed, ligation of the right common carotid artery and internal jugular vein didn't cause ipsilateral cerebral hemisphere injury. The data from the UK group demonstrated that an exact survival improvement was observed in neonates receiving ECMO support than those treated with conventional management (2). The RESTORE study also made a negative conclusion in pediatric patients treated with ECMO comparing with non ECMO support patients. According to the hypothesis from Cashen et al., survivors have got a worse neurocognitive result after treating with ECMO (29). However, the results from our study do not support the hypothesis. For neonates with ARDS, either the primary disease or the ECMO treatment could be a risk factor for the damage to the neonatal central nervous system (30). The neonates in this study would be followed long-term to evaluate their neurodevelopment.

It has been demonstrated that ECMO can decrease the mortality in neonates with respiratory failure (2). After the publication of the Montreux definition, it was well-known that ARDS was one special type of neonatal respiratory failure. In this study, we enrolled neonates who developed severe neonatal ARDS (OI≥16) and needed invasive ventilation after birth. However, only when the neonates sustained an OI≥40 for at least 4 h could they be considered ECMO candidates. We found superior outcomes in ECMO-supported vs. non-ECMO-supported neonates with ARDS of OI≥40.

However, there are still some limitations in the study. Firstly, the sample size is relatively small. Although efforts are made for matching, there are still differences in the levels of PaO_2_ and lactate before ECMO support. Secondly, because the pediatric ECMO technology has just started in mainland China, only two of the five centers were equipped with neonatal ECMO technology. Thirdly, as propensity score matching has been used to evaluate without a true randomized, controlled trial, it does not match unmeasured confounders. The decision for a patient to be or not to be on ECMO is often not captured and confounding from these issues cannot be adequately controlled.

## Conclusions

For neonates with severe ARDS(OI≥40), our results demonstrate that support with ECMO could receive a better clinical outcome compared with the conventional treatment without prolonged ventilator-time or ICU stay. However, there is still necessity for a rigorous multi-center randomized controlled clinical trial for the efficiency of ECMO in several neonatal HRF to provide definitive conclusions.

## Data Availability Statement

The datasets generated for this study are available on request to the corresponding author.

## Ethics Statement

The studies involving human participants were reviewed and approved by The Ethics Committee's examination and approve document of the Army General Hospital. Written informed consent to participate in this study was provided by the participants' legal guardian/next of kin. Written informed consent was obtained from the minor(s)' legal guardian/next of kin for the publication of any potentially identifiable images or data included in this article.

## Author Contributions

All authors listed have made a substantial, direct and intellectual contribution to the work, and approved it for publication.

## Conflict of Interest

The authors declare that the research was conducted in the absence of any commercial or financial relationships that could be construed as a potential conflict of interest.
